# Development of a hemodialysis safety checklist using a structured panel process

**DOI:** 10.1186/s40697-015-0039-8

**Published:** 2015-02-12

**Authors:** Samuel A Silver, Alison Thomas, Andrea Rathe, Pamela Robinson, Ron Wald, Ziv Harel, Chaim M Bell

**Affiliations:** Division of Nephrology, St. Michael’s Hospital, University of Toronto, Toronto, Canada; Department of Medicine and Keenan Research Center, Li Ka Shing Knowledge Institute of St Michael’s Hospital, University of Toronto, Toronto, Canada; Institute of Health Policy, Management, and Evaluation, University of Toronto, Toronto, Canada; Department of Medicine, Mount Sinai Hospital, University of Toronto, Toronto, Canada

**Keywords:** Checklist, Delphi panel, Hemodialysis, Patient safety, Quality improvement

## Abstract

**Background:**

The World Health Organization created a Surgical Safety Checklist with a pause or “time out” to help reduce preventable adverse events and improve communication. A similar tool might improve patient safety and reduce treatment-associated morbidity in the hemodialysis unit.

**Objective:**

To develop a Hemodialysis Safety Checklist (Hemo Pause) for daily use by nurses and patients.

**Design:**

A modified Delphi consensus technique based on the RAND method was used to evaluate and revise the checklist.

**Setting:**

University-affiliated in-center hemodialysis unit.

**Participants:**

A multidisciplinary team of physicians, nurses, and administrators developed the initial version of the Hemo Pause Checklist. The evaluation team consisted of 20 registered hemodialysis nurses.

**Measurements:**

The top 5 hemodialysis safety measures according to hemodialysis nurses. A 75% agreement threshold was required for consensus.

**Methods:**

The structured panel process was iterative, consisting of a literature review to identify safety parameters, individual rating of each parameter by the panel of hemodialysis nurses, an in-person consensus meeting wherein the panel refined the parameters, and a final anonymous survey that assessed panel consensus.

**Results:**

The literature review produced 31 patient safety parameters. Individual review by panelists reduced the list to 25 parameters, followed by further reduction to 19 at the in-person consensus meeting. The final round of scoring yielded the following top 5 safety measures: 1) confirmation of patient identity, 2) measurement of pre-dialysis weight, 3) recognition and transcription of new medical orders, 4) confirmation of dialysate composition based on prescription, and 5) measurement of pre-dialysis blood pressure. Revision using human factors principles incorporated the 19 patient safety parameters with greater than or equal to 75% consensus into a final checklist of 17-items.

**Limitations:**

The literature review was not systematic. This was a single-center study, and the panel lacked patient and family representation.

**Conclusions:**

A novel 17-item Hemodialysis Safety Checklist (Hemo Pause) for use by nurses and patients has been developed to standardize the hemodialysis procedure. Further quality improvement efforts are underway to explore the feasibility of using this checklist to reduce adverse events and strengthen the safety culture in the hemodialysis unit.

**Electronic supplementary material:**

The online version of this article (doi:10.1186/s40697-015-0039-8) contains supplementary material, which is available to authorized users.

## Abrégé

**Contexte:**

L’Organisation mondiale de la Santé a mis au point une liste de contrôle de la sécurité chirurgicale offrant ainsi la possibilité de faire une pause ou de « prendre du temps » pour réduire les effets indésirables qui sont prévisibles et pour améliorer la communication. Un outil similaire pourrait améliorer la sécurité du patient et diminuer la morbidité associée au traitement sur un service d’hémodialyse.

**Objectif de l’étude:**

Mettre au point une liste de contrôle de la sécurité en hémodialyse (Hemo Pause) pour utilisation quotidienne, à l’intention des infirmières et des patients.

**Type d’étude:**

L’évaluation et la révision de la liste de contrôle ont été effectuées avec une version modifiée de l’outil de construction de consensus Delphi, basée sur la méthode RAND.

**Contexte de l’étude:**

Service de dialyse affilié à un centre universitaire.

**Participants:**

La version initiale de la liste de contrôle de sécurité Hemo Pause a été mise au point par une équipe multidisciplinaire comprenant médecins, infirmières et personnel administratif. L’équipe d’évaluation de l’outil était formée de 20 infirmières autorisées œuvrant en hémodialyse.

**Mesures:**

Les 5 mesures de sécurité les plus importantes, selon les infirmières en hémodialyse. Un seuil d’entente de 75% était requis pour le consensus.

**Méthodes:**

La méthode d’appel au panel s’est effectuée de façon itérative. Celle-ci consistait en: une revue de la littérature afin de faire ressortir les paramètres de sécurité; l’attribution, par le panel d’infirmières en hémodialyse, d’une valeur pour chacun des paramètres; une réunion de concertation de groupe durant laquelle le panel a raffiné les paramètres, et, finalement, d’un questionnaire anonyme visant à évaluer le consensus atteint par le panel.

**Résultats:**

La revue de la littérature a fait ressortir 31 paramètres de sécurité des patients. La revue individuelle faite par les membres du panel a réduit cette liste à 25 paramètres, puis celle-ci a été de nouveau réduite à 19 lors de la réunion de concertation de groupe. L’évaluation finale s’est soldée sur les 5 mesures de sécurité les plus importantes suivantes: 1) la confirmation de l’identité du patient; 2) la mesure du poids du patient avant la dialyse; 3) la vérification de nouvelles prescriptions et leur retranscription; 4) la confirmation de la composition du dialysat avec la prescription, et 5) la mesure de la pression artérielle avant la dialyse. Utilisant des principes concernant des facteurs humains, une révision des 19 paramètres de sécurité des patients avec un consensus plus grand ou égal à 75% a permis d’établir une liste de contrôle finale de 17 items.

**Limites de l’étude:**

La revue de la littérature n’était pas systématique. Cette étude s’est déroulée en un seul centre; de plus, le panel ne représentait ni le patient ni sa famille.

**Conclusions:**

Une liste de contrôle de la sécurité en hémodialyse (Hemo Pause) a été mise au point afin de standardiser les traitements d’hémodialyse. Des efforts poussés d’amélioration de la qualité sont en branle afin d’explorer la faisabilité de l’utilisation de cette liste de contrôle innovatrice pour réduire les occurrences d’événements indésirables, et renforcer la vigilance sur les services d’hémodialyse.

## What was known before

Errors during in-center hemodialysis are common, and patients often worry that an error will occur during their hemodialysis session. Checklists are a useful patient safety strategy that have improved care across a number of medical disciplines.

## What this adds

The first safety checklist created specifically for the hemodialysis procedure, using a proven technique for developing quality and patient safety measures in healthcare. This provides other dialysis facilities with an example to produce their own patient safety tools, and may catalyze local quality improvement efforts to enhance the safety of dialysis patients.

## Background

The *“To Err is Human”* report published by the Institute of Medicine estimated that 98,000 patients die each year from preventable medical errors in the United States [1]. Similar rates of preventable adverse events have been reported in Canada [2], Australia [3], New Zealand [4,5], and England [6]. The key lesson from these studies is that errors are usually a result of the system of care, rather than the individual.

Patients with end-stage renal disease (ESRD) are at high risk for medical errors given their frequency of complex treatment, comorbidities, polypharmacy, physiological consequences of ESRD, and coordination with other hospital departments to provide care [7,8]. These factors all contribute to a stressful and busy hemodialysis (HD) unit, so it is not surprising that medical errors are common in the ESRD population.

A 2006 survey documented provider and patient opinions on safety in the HD unit [9]. Frequent problems included greater than 2 vascular access needle insertion attempts (30%), access clotting (20%), machine difficulties leading to early treatment stoppage (15%), failure to record pre-dialysis blood pressure or weight (13%), and access needle disconnection (5%) [9]. Almost half (49%) of the patients who responded indicated that they are sometimes or always worried that a mistake will occur during their HD treatment [9].

Subsequent studies have yielded further insight into the unique HD safety issues. A review of 526 HD incident reports to the Pennsylvania Patient Safety Authority over a 12 month period identified medication errors (29.0%), protocol violations (12.9%), and falls (5.9%) as common errors during HD treatment [8]. Of these events, 87.6% reached the patient, and 5.5% resulted in patient harm [8]. More recently, a Scottish retrospective study found that in 3.5% of ESRD patient deaths there was an area of concern identified that likely contributed to the death, and 2.1% of patient deaths were a direct result of a dialysis complication [7]. Given the frequency and severity of errors associated with dialysis, multiple experts have called for action [10,11].

Checklist utilization is one patient safety strategy that may be effective. Checklists have been shown to improve patient safety, adherence to protocols/policies, communication, teamwork, and consistency of care by standardizing procedures [12]. The pause or “time out” discussion prior to an invasive procedure is an important component [12]. Checklists have been effective across several disciplines, most notably surgery and central line insertion [13,14]. The HD unit may be a particularly appropriate venue for a safety checklist, where it may prevent errors under stressful conditions and maintain staff precision, focus, clarity, and memory recall [15].

At present, a HD safety checklist does not exist. Therefore, our objective was to develop a HD safety checklist using a structured, consensus-based, panel process. Our focus was to develop a checklist that would improve consistency of care and provider/patient communication, as well as be feasible to implement in clinical practice.

## Methods

### Panel members

The checklist development team consisted of 5 members, including a nephrologist, Nephrology fellow, nurse practitioner, nurse administrator, and researcher. Both nursing professionals are experienced HD nurses. This team created the pilot checklist and Delphi panel review materials. Members were selected as a result of their shared interests in patient safety, quality improvement, experience as frontline HD staff, and related research initiatives. The checklist evaluation team consisted of 20 registered HD nurses. This latter group completed the Delphi panel process to assess the content and feasibility of the HD safety checklist. Frontline hemodialysis staff were involved throughout the process to ensure ongoing input from the intended end-users of the Hemo Pause checklist, which is an important component of successful quality improvement [16].

### Initial work: literature review and checklist development

A literature review was conducted to inform the content and format of the HD safety checklist. The development team searched PubMed for relevant articles using a search strategy that has been previously described by the Renal Physicians Association (RPA) [17]. Since our literature review was targeted rather than systematic, we did not track the number of articles screened or reviewed. Bibliographies of all relevant articles were reviewed to identify additional studies. In addition, we conducted a focused study of select resources, such as the RPA survey [9] and national HD guidelines [18-20]. Available toolkit resources were examined in detail, including those developed by the RPA [21], the Forum of End Stage Renal Disease Networks [22], and the Agency for Healthcare Research and Quality [23].

The development team used the results of this literature review to identify patient safety parameters that could be considered for inclusion in a HD safety checklist. A list of these parameters was provided to A.T, a nurse practitioner with over 30 years of HD experience, who created the first version of the checklist. The development team then met 3 times in person between February 2013 and June 2013. At the first meeting, the team reviewed the list of patient safety parameters, evidence-based recommendations and patient safety toolkits around best HD practices that were identified by the literature review. When evidence was lacking, the development team voted on whether an item would be included on the checklist. The second meeting provided an opportunity for individual comments and feedback on checklist design, which was guided by examples from the literature review. It was decided to model the checklist after the World Health Organization (WHO) surgical safety checklist [13], including a pause or “time out” before connecting the patient to the HD machine. A final meeting ensured consensus of the development team on every element of the Hemo Pause Checklist. This version was presented to the evaluation panel. The Hemo Pause quality improvement initiative was approved by both the medical and nursing leadership of the in-center HD unit at St. Michael’s Hospital in Toronto, Canada.

### Overview of modified Delphi panel process

**Figure 1:**
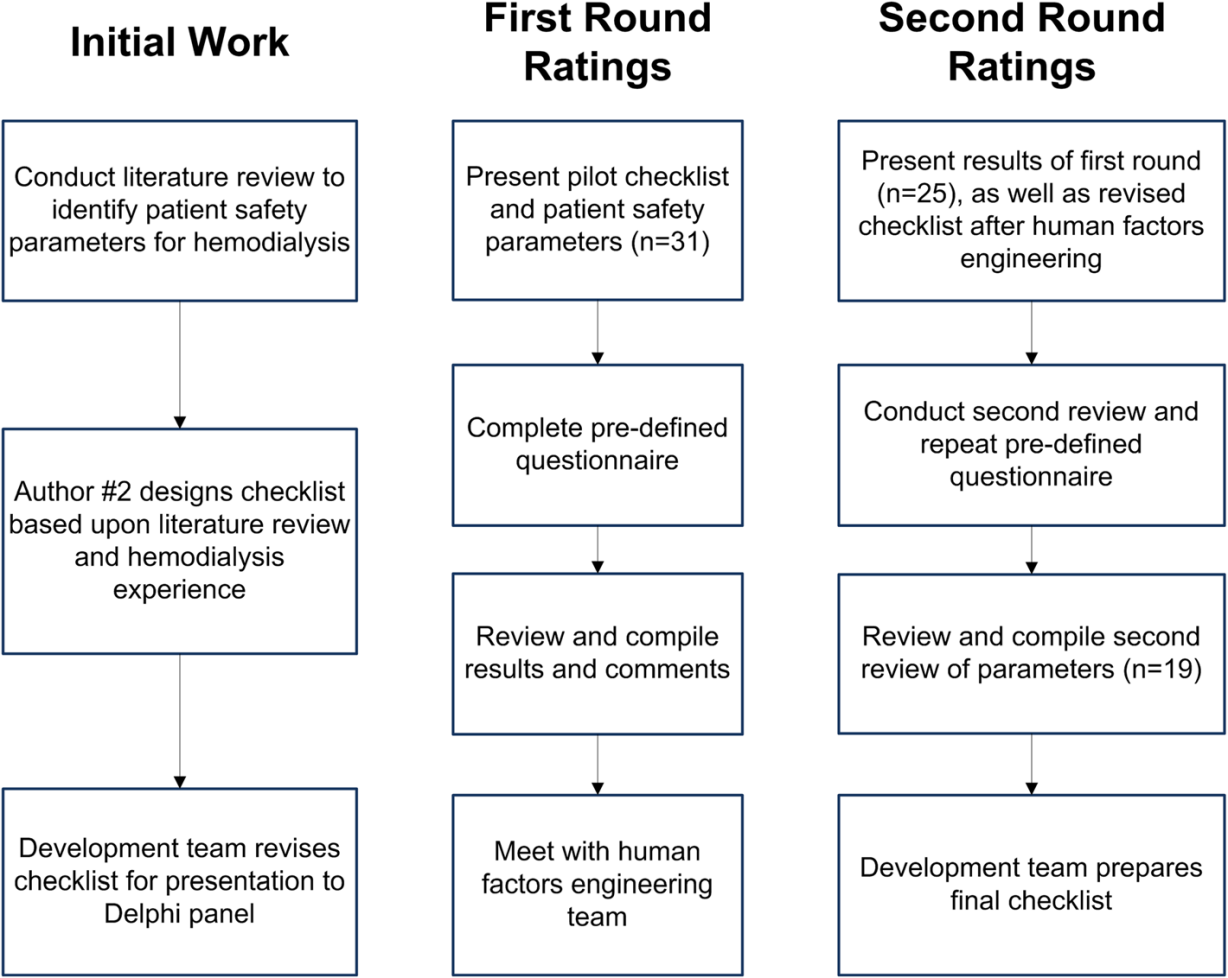
**Illustration of modified Delphi process.**

A modified Delphi consensus technique, based on the RAND method, was used [24]. We followed a structured process through which the expertise and knowledge of a group of individuals was systematically obtained through questionnaires interspersed by opinion feedback. For this study, multiple stages of the modified Delphi technique (literature review, individual rating, face-to-face consensus meeting, and final ranking) were used to allow for optimal, unbiased expression of opinions (Figure 1). This is a proven technique for developing quality and patient safety measures in healthcare [25-32].

### Delphi panel ratings

In the first round, an information session was held to introduce the evaluation team to the Hemo Pause Checklist and the Delphi process. The participants were asked to anonymously answer predefined standardized questions that comprised 5 sections (Additional file 1: Figure S1).

Section 1 presented 31 patient safety parameters that were identified by the literature review and asked the panel which must be addressed to complete a safe HD session (Table 1). Section 2 asked the panel to rank the 5 most important patient safety parameters from the list of 31 parameters. At this point, the panel was shown the pilot version of the Hemo Pause Checklist (Additional file 2: Figure S2). Section 3 listed the patient safety parameters from section 1 that were not included in the checklist, and asked the panel if any should be added. Section 4 asked the panel to rate the checklist design from a scale of 1 to 5, with 1 representing very poor, 3 average, and 5 excellent. Section 5 asked the panel if there were any barriers that might interfere with checklist completion.

**Table 1:** **Patient safety parameters and Delphi panel results**

**Patient safety parameter**	**% of panel selecting parameter**	
	***First round***	***Second round***
Patient identification	**100**	**100**
Pre-dialysis weight	**95**	**100**
Allergies checked	**95**	**95**
Doctor’s orders noted and transcribed	**95**	**95**
Medications correctly administered	**100**	**85**
Treatment plan reviewed with patient	**90**	70
Patient asked about health concerns	**100**	**85**
Hand-washing	**90**	**85**
Pre-dialysis blood pressure	**95**	**100**
Easy cannulation	65	65
Correct needle insertion	**80**	**80**
Pain-free cannulation	60	20
Secured dialysis needles	**85**	70
Correct dialyzer	**100**	**95**
Correct dialysis solution	**95**	**100**
Correct machine setting	**85**	**100**
No circuit clotting	**75**	70
Blood pump speed at the prescribed rate	**80**	**75**
Blood samples collected	**80**	**80**
Blood specimens correctly labeled	**95**	**90**
Dialysis treatment for complete duration	70	45
No blood loss	**80**	70
Blood clotting after dialysis	**75**	65
No evidence of access infection	**85**	**80**
Post-dialysis blood pressure	**95**	**100**
Post-dialysis weight	**90**	**100**
No patient falls	**80**	**80**
No needle stick injuries	55	25
Medical errors reported if witnessed	60	40
Management support for incident reporting	70	40
Adherence to procedures	**80**	70

*Bold font indicates a parameter with ≥75% consensus.

Prior to the second round of the Delphi panel, the checklist was revised based on the first round results. It was modified using recommended human factors engineering concepts by Healthcare Human Factors at Toronto General Hospital [33]. Human factors engineering attempts to identify and address safety problems that arise due to the interaction between people, technology, and work environments [33].

During the second round, all panel members met in person for a 1-day structured discussion of the results from the first round. At the conclusion of the discussion, members were asked to complete a second round of the questionnaire. Individual results remained anonymous to other panel members. After completion of all discussion and questionnaires, panelists were asked to comment upon the overall consensus process.

### Data analysis

Consensus on the questionnaires was defined as ≥75% agreement amongst panel members (standard percentage in other studies) [29,30,32]. The top 5 patient safety parameters were calculated using a weighted frequency. A top 5 factor received between 5 points and 1 point, based on the number assigned by the panel. The weighted frequency was calculated as a sum of all the points received, with a maximum score of 100 points indicative of the most important.

## Results

### Checklist development

Table 1 lists the 31 patient safety parameters identified by the literature review. The pilot version of the checklist presented at the first Delphi panel meeting is illustrated in a supplementary figure (Additional file 2: Figure S2).

### First round ratings

From the initial set of 31 safety parameters, 25 received an aggregate rating of ≥75% (Table 1). The top 5 safety measures were: 1) confirmation of patient identity (99/100), 2) measurement of pre-dialysis weight (39/100), 3) recognition and transcription of new medical orders (38/100), 4) confirmation of dialysate composition based on prescription (38/100), and 5) measurement of pre-dialysis blood pressure (11/100). The checklist design received a mean score of 3.75/5.

### Checklist revisions

The following patient safety parameters were added to the checklist based upon the first round results: patient identification, patient allergies, and pre-dialysis blood pressure. Human factors engineering helped to clarify tasks, remove duplication, and shorten text. They recommended that hand-washing be removed from the checklist, since it could not be easily incorporated due to the multiple times a provider must wash their hands during a HD session. Therefore, its inclusion on the checklist at only one time point would be incorrect, but its inclusion at multiple time points would be space-consuming and cumbersome. The layout was also modified to be more consistent with the WHO Surgical Safety Checklist [13].

### Second round ratings

Analysis from the second round of panel ratings revealed a set of 19 parameters that received an aggregate rating of ≥75% (Table 1). All parameters with ≥75% consensus were included in the final checklist except hand-washing, correct needle insertion, and falls. These were excluded due to human factors concerns, since they were not amenable to a single and clear checklist action. The top 5 safety measures were unchanged from the first round. The relative weighting was 1) confirmation of patient identity (95/100), 2) measurement of pre-dialysis weight (58/100), 3) recognition and transcription of new medical orders (35/100), 4) confirmation of dialysate composition based on prescription (32/100), and 5) measurement of pre-dialysis blood pressure (10/100). The checklist design received a mean score of 4/5 (P = 0.09, compared to the pilot checklist, Additional file 2: Figure S2). Frequent barriers to checklist implementation were duplication of work and time pressures, with 95% and 85% panel agreement respectively.

A post-panel survey found unanimous agreement from the participants that their opinions were valued, the process was fair, and that no one changed their answers as a result of intimidation.

### Final checklist

**Figure 2:**
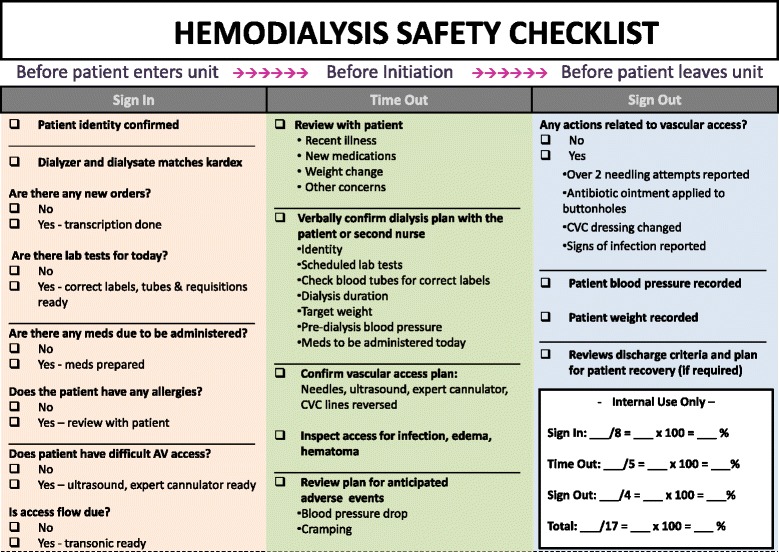
**Final version of Hemodialysis Safety Checklist following Delphi panel process.** CVC = central venous catheter.

Figure 2 demonstrates the final version of the Hemo Pause Checklist. The development team unanimously decided to include items on HD access, duration, and anticipated adverse events, despite these items not receiving ≥75% consensus because of their strong evidentiary basis or clinical face validity. This yielded a final checklist of 17-items.

## Discussion

Through a structured Delphi panel process, a HD safety checklist (Hemo Pause) was developed using evidence-based patient safety parameters and frontline staff experience. The decision to evaluate the checklist with a panel of HD nurses strengthened the utility of the document, since the intention of the checklist was for it to be used routinely by nurses and patients during HD. This approach helped ensure that the selected patient safety parameters not only adhered to the general principles of being scientifically sound, but also were clinically relevant and feasible to implement.

The final list of 19 patient safety parameters that received ≥75% consensus included actions from different time points of the HD procedure. Several attempts were made by the development team to incorporate all of these parameters into the final checklist. However, human factors engineering concepts limited the inclusion of some parameters (hand-washing, correct needle insertion, and falls). As such, some parameters were excluded to ensure that checklist tasks were clear and could be properly integrated by frontline staff into their normal workflow [33].

The feasibility of widely applying the HD safety checklist was a prime consideration. It was designed to be used collaboratively by nurses and patients at every HD session. Not surprisingly, duplication of work and time pressures were perceived by the panel as barriers to checklist implementation, even prior to piloting the checklist with patients. Several nurses also commented that many of the checklist items are already completed as part of usual practice. This is not surprising, since one of the purposes of a safety checklist is to standardize usual care, providing a barometer from which to measure errors and identify quality gaps. While these workload concerns can only be established by piloting the checklist in real practice, these observations underscore the importance of context in patient safety and quality improvement [16,34]. Frontline staff noted that the checklist needed to provide them with an immediate advantage, either in the form of reduced documentation or improved patient satisfaction in order to offset the upfront time investment required for checklist completion. These deliverables to frontline staff will be needed to sustain future checklist quality improvement efforts.

To our knowledge, this is the first reported safety checklist that is uniquely geared to HD treatment. In a systematic review of published renal replacement therapy quality improvement initiatives, none of the 93 studies focused on patient safety [35]. This is an important gap that must be filled. Previous safety efforts in dialysis have focused on measuring the rate of preventable adverse events, primarily involving voluntarily surveys [9] or incident reporting [8]. Reporting bias is a clear limitation to these methods, with variations over time more likely to reflect changes in reporting patterns than changes in patient safety [36]. A recent study by Bray et al. retrospectively reviewed all dialysis deaths over a 3.5 year period to identify preventable factors that may have contributed to mortality [7]. They identified preventable factors that may have or did contribute to death in 3.5% of deaths. These included errors related to communication, organization, and human factors, and were due to five main causes: management of hyperkalemia, prescribing, out of hours care, infection, and vascular access. An accompanying editorial called upon nephrologists “to take clearly articulated steps to improve the safety of the patients who trust them to provide care [11]”. Examples listed included standardization, teamwork, and culture change.

Our Hemo Pause Checklist may be able to improve all of these components. Checklists provide a reminder and cognitive aid for tasks, such as the multistep HD procedure [15]. They promote teamwork and communication, such as those between dialysis nurses and patients [12], which is the purpose of the pause or “time out” discussion before connecting the patient to the HD machine. Lastly, checklists highlight the importance of patient safety, where errors represent a failure of the system rather than an individual. Despite these advantages, the Hemo Pause Checklist should not be viewed as a universal solution. The limitations of checklists in medicine have been well documented, and include over-reliance, non-adherence, and implementation challenges [15,37,38]. A recent study of mandatory WHO Surgical Checklist use in Ontario, Canada demonstrated no province-wide effect on morbidity and mortality, suggesting different adherence and implementation strategies between the 101 hospitals [38,39]. On the other hand, the success of the Michigan Keystone Project on reducing rates of catheter-related bloodstream infection by 66% in 108 intensive care units was not only the result of a checklist, but also due to the system change and frontline engagement that accompanied its adoption [14,37,38].

Therefore, the Hemo Pause Checklist must be piloted in the HD unit using quality improvement and change management methods before it can be spread more widely. This will allow for the determination of checklist feasibility, measurement of performance gaps between checklist and usual care, and local checklist modifications to suit the patient safety concerns and workflow of individual HD units. We have initiated such a study in our dialysis unit, and encourage others to evaluate Hemo Pause in their local environments or undertake a structured panel process to develop their own patient safety tools.

Some limitations to our checklist development and modified Delphi panel process require discussion. First, the review of literature was not a systematic review, rather it was a targeted review designed by the development team. While it is possible that some evidence-based safety parameters were missed, this is unlikely given the paucity of literature that was identified. Second, the lack of patient safety literature in dialysis means that individual items on the checklist may not have an extensive evidence base; however, all elements on the checklist have clinical face validity. Third, the Delphi panel consisted only of HD nurses at a single-center to maximize feedback from the frontline workers for whom the checklist was designed. Although this excluded other important perspectives and may limit generalizability, the HD treatment constitutes a fairly stereotyped process with much similarity among different HD units. Many successful checklists have been designed in single-centers, including those from the Michigan Keystone Project [40-43]. Moreover, to minimize selection bias, nephrologists and administrators reviewed the final version of the checklist, and included 3 parameters (HD access, duration, and anticipated adverse events) that did not reach ≥75% panel consensus but had either a strong evidentiary basis or clinical face validity. We were unable to obtain patient and caregiver representation on either the development or evaluation team. Fourth, the standardized questions contained a large number of variables available for selection. While it was imperative to include all of the major patient safety parameters that emerged from the literature review, this has the potential to compromise the accuracy of the Delphi method [44]. This could have contributed to a “serial position effect”, meaning that the first factors listed were treated differently than factors listed further down [45]. We attempted to minimize this effect by ordering the parameters according to their temporal relationship in the HD procedure. Lastly, the potential for strong personalities and opinions to dominate the direction of discussion is always a concern. To combat this, we completed the anonymous survey twice to ensure that each participant had an opportunity to express their view. The final survey confirmed that all participants were satisfied with the process.

Our findings have important implications. Firstly, it highlights parameters that are needed for a safe HD session, both according to the literature and a multidisciplinary team of Nephrology personnel. Secondly, it outlines the first HD safety checklist to standardize care and communication in the HD unit. Lastly, our study identifies barriers to checklist implementation to inform patient safety and quality improvement efforts.

## Conclusions

Patient safety is an emerging field, and there are many opportunities in Nephrology for improvement. The modified Delphi consensus panel approach applied in this study enabled us to develop and evaluate the first HD safety checklist (Hemo Pause). This methodology and the multidisciplinary representation provide strong face validity to the patient safety parameters included on the checklist. We believe that these findings provide other dialysis facilities with a roadmap to produce patient safety tools and catalyze local quality improvement efforts to enhance the health and safety of dialysis patients.

More research and testing of dialysis safety measures are clearly needed to determine their usefulness and feasibility. At our center, quality improvement efforts are underway to answer these questions, as well as determine if the Hemo Pause Checklist can reduce adverse events and strengthen the safety culture in the HD unit.

## Additional files

Additional file 1: Figure S1. Standardized questionnaire presented to Delphi panel. Additional file 2: Figure S2. Pilot version of the checklist presented at the first Delphi panel meeting. MAR = medication administration record, N/S = normal saline, U/S = ultrasound, CVC = central venous catheter, AVF = arteriovenous fistula, AVG = arteriovenous graft, VAC = vascular access committee, NP = nurse practitioner, BP = blood pressure.

## Abbreviations

ESRD: End-stage renal disease
HD: Hemodialysis
RPA: Renal physicians association
WHO: World Health Organization
