# Supplementary material for: Development of a hemodialysis safety checklist using a structured panel process
Source: Can J Kidney Health Dis. 2015 Feb 12;2:5. doi: 10.1186/s40697-015-0039-8 (PMC4349476; doi:10.1186/s40697-015-0039-8)
Supplement: Additional file 1: Figure S1. — Standardized questionnaire presented to Delphi panel. [file 40697_2015_39_MOESM1_ESM.docx]

# Additional file 1: Figure S1: Standardized questionnaire presented to Delphi panel Section 1: What is required for a safe dialysis session? (check all that apply)

|            | Patient identification Pre-dialysis weight Allergies checked  Doctor’s orders noted and transcribed Medications correctly administered  Treatment plan reviewed with patient |            | No circuit clotting  Blood pump speed at the prescribed rate Blood samples collected  Blood specimens correctly labelled Dialysis treatment for complete duration  No blood loss |
| --- | --- | --- | --- |
|  | Patient asked about health concerns |  | Blood clotting after dialysis |
|  | Hand washing |  | No evidence of access infection |
|  | Pre-dialysis blood pressure |  | Post-dialysis blood pressure |
|  | Easy cannulation |  | Post-dialysis weight |
|  | Correct needle insertion |  | No patient falls |
|  | Pain-free cannulation |  | No needle stick injuries |
|  | Secured dialysis needles |  | Medical errors reported if witnessed |
|  | Correct dialyzer |  | Management support for incident reporting |
|  | Correct dialysis solution |  | Adherence to procedures |
|  | Correct machine setting |  |  |

Other (please list):

# Section 2: From the parameters you have chosen, which are the five most important? (1=most important)

**Section 3: Now that you have seen the Hemodialysis Safety Checklist, are there any items that you would add to it?**

- Pre-dialysis weight
- Allergies checked
- Patient asked about health concerns
- Pre-dialysis blood pressure
- Correct needle insertion
- Pain-free cannulation
- Secured dialysis needles
- No circuit clotting
- Blood pump speed at the prescribed rate
- Dialysis treatment for complete duration
- No blood loss
- Blood clotting after dialysis
- No patient falls
- Medical errors reported if witnessed

Other (please list):

# Section 4: How would you rate the current checklist design?

Excellent [ ] Above average [ ] Average [ ] Below average [ ] Very poor [ ]

Are there any changes you would make to the current checklist design? (consider layout, color, font, fill in the blanks instead of checkmarks)

# Section 5: Identify any barriers that would prevent you from completing the checklist (check all that apply)

- Time
- Duplicates work I already do
- Too long
- Too complicated
- Poor layout
- Will not be effective
- I do not believe in checklists
- Patients will not participate

Other (please list):
