# Supplementary material for: Development of a hemodialysis safety checklist using a structured panel process
Source: Can J Kidney Health Dis. 2015 Feb 12;2:5. doi: 10.1186/s40697-015-0039-8 (PMC4349476; doi:10.1186/s40697-015-0039-8)
Supplement: Additional file 2: Figure S2. — Pilot version of the checklist presented at the first Delphi panel meeting. MAR = medication administration record, N/S = normal saline, U/S = ultrasound, CVC = central venous catheter, AVF = arteriovenous fistula, AVG = arteriovenous graft, VAC = vascular access committee, NP = nurse practitioner, BP = blood pressure. [file 40697_2015_39_MOESM2_ESM.pptx]

## Slide 1
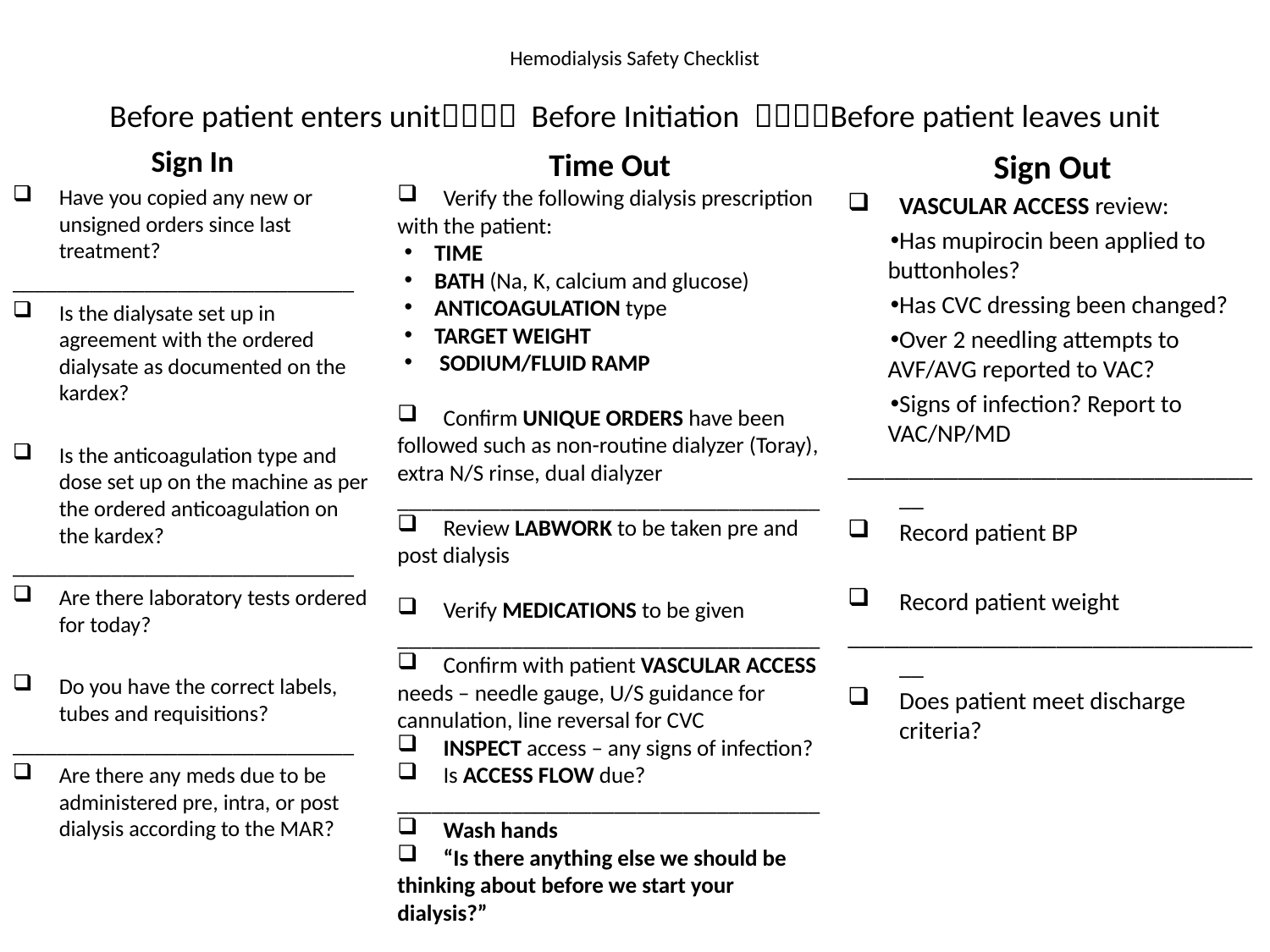

# Hemodialysis Safety Checklist
Before patient enters unit Before Initiation Before patient leaves unit
Sign In
Have you copied any new or unsigned orders since last treatment?
_______________________________
Is the dialysate set up in agreement with the ordered dialysate as documented on the kardex?
Is the anticoagulation type and dose set up on the machine as per the ordered anticoagulation on the kardex?
_______________________________
Are there laboratory tests ordered for today?
Do you have the correct labels, tubes and requisitions?
_______________________________
Are there any meds due to be administered pre, intra, or post dialysis according to the MAR?
Time Out
 Verify the following dialysis prescription with the patient:
TIME
BATH (Na, K, calcium and glucose)
ANTICOAGULATION type
TARGET WEIGHT
 SODIUM/FLUID RAMP
 Confirm UNIQUE ORDERS have been followed such as non-routine dialyzer (Toray), extra N/S rinse, dual dialyzer
_____________________________________
 Review LABWORK to be taken pre and post dialysis
 Verify MEDICATIONS to be given
_____________________________________
 Confirm with patient VASCULAR ACCESS needs – needle gauge, U/S guidance for cannulation, line reversal for CVC
 INSPECT access – any signs of infection?
 Is ACCESS FLOW due?
_____________________________________
 Wash hands
 “Is there anything else we should be thinking about before we start your dialysis?”
Sign Out
VASCULAR ACCESS review:
Has mupirocin been applied to buttonholes?
Has CVC dressing been changed?
Over 2 needling attempts to AVF/AVG reported to VAC?
Signs of infection? Report to VAC/NP/MD
___________________________________
Record patient BP
Record patient weight
___________________________________
Does patient meet discharge criteria?
